# *Poria cocos* Polysaccharide-Modified Selenium Nanoparticles: Structural Characterization, Stability, and In Vitro Antioxidant and Anti-Inflammatory Activity Studies

**DOI:** 10.3390/foods14203555

**Published:** 2025-10-18

**Authors:** Tao Shu, Fan Li, Jiang-Ning Hu, Yu Xu

**Affiliations:** 1 National Key Laboratory for Development and Utilization of Forest Food Resources, Zhejiang A&F University, Hangzhou 311300, China; 2 College of Food and Health, Zhejiang A&F University, Hangzhou 311300, China; 3 SKL of Marine Food Processing & Safety Control, National Engineering Research Center of Seafood, Collaborative Innovation Center of Seafood Deep Processing, School of Food Science and Technology, Dalian Polytechnic University, Dalian 116034, China

**Keywords:** *Poria cocos* polysaccharides, selenium nanoparticles, stability, antioxidant activity, anti-inflammatory activity

## Abstract

Selenium nanoparticles (Se NPs) have received increasing attention as a new alternative source to other forms of selenium in nutritional dietary supplements; however, the limited stability and pronounced tendency of selenium nanoparticles (Se NPs) to aggregate in aqueous environments have significantly constrained their practical applications. In this study, *Poria cocos* polysaccharide-modified Se NPs (PCP-Se NPs) were synthesized by the selenite/ascorbic acid chemical reduction method. PCP-Se NPs exhibited a uniformly dispersed spherical morphology with an average particle size of 66.64 ± 0.30 nm, and displayed an amorphous crystal structure. Compared to unmodified Se NPs, the PCP-Se NPs exhibited low Se release (8.83 ± 0.73%) after simulated gastrointestinal digestion, and they had excellent storage stability and salt ion stability. PCP-Se NPs exhibited potent antioxidant activity manifested by the effective scavenging of DDPH and ABTS radicals. PCP-Se NPs were efficiently internalized by RAW264.7 cells and released into the cytoplasm by a lysosomal escape mechanism, thereby effectively reducing intracellular inflammatory factor levels (the levels of MPO, NO, iNOS, TNF-α, IL-1β, and IL-10 in the PCP-Se NPs treatment group were 0.38 ± 0.013-fold, 0.26 ± 0.02-fold, 0.36 ± 0.02-fold, 0.57 ± 0.03-fold, 0.35 ± 0.02-fold, and 2.07 ± 0.16-fold that of the LPS group, respectively), alleviating oxidative stress (the levels of CAT, SOD, GSH, and MDA in the PCP-Se NP-treated group were 2.48 ± 0.02-fold, 1.91 ± 0.11-fold, 3.16 ± 0.28-fold, and 0.46 ± 0.03-fold that of the LPS group, respectively), and maintaining mitochondrial membrane potential stability. This study provides a basis and reference for improving the stability of Se NPs and developing novel selenium-enriched dietary supplements.

## 1. Introduction

Selenium (Se) serves as a vital micronutrient and constitutes the catalytic core of numerous enzymes, thereby fulfilling an indispensable functional role in a variety of physiological processes such as maintaining the balance of redox reactions in the body, preventing disease and enhancing immune function [1]. Selenium deficiency may lead to decreased immunity, viral infections, thyroid dysfunction, oxidative stress, inflammation and cardiovascular disease [2]. In view of this, according to the World Health Organization (WHO), adults require a daily intake of 55 to 400 micrograms of selenium to sustain essential physiological functions and support overall health maintenance [3]. However, there is a small gap between the beneficial dose and toxicity of Se, resulting in its extremely limited practical use in dietary supplements. Moreover, due to their high chemical reactivity, oxidized forms of organic or inorganic selenium such as selenate (SeO_4_^2−^, +6), selenite (SeO_3_^2−^, +4), and selenides (H_2_Se, −2) used to supplement selenium are highly toxic, limiting their clinical application [4]. Therefore, there is a need to find a new source of selenium dietary supplements with higher safety and biocompatibility.

Owing to advancements in nanotechnology, selenium nanoparticles (Se NPs) have garnered substantial research interest in recent years due to their favorable attributes, including minimal toxicity, enhanced bioavailability, efficient biodegradability, and superior biocompatibility [5]. More importantly, Se NPs have significant antioxidant, anti-inflammatory and anti-tumor activities, and have been used in the treatment of a wide range of diseases, including hepato-renal toxicity, inflammatory disorders, cancer, diabetes and cardiovascular diseases [6]. For instance, Chen et al. [7] demonstrated that selenium nanoparticles suppress oxidative stress and inflammation in cardiomyocytes by inhibiting STAT1. Liu et al. [8] confirmed that selenium nanoparticles inhibit drug-free recurrence in cervical cancer by suppressing ABC transporter expression. Nevertheless, owing to their limited stability and elevated surface energy, selenium nanoparticles (Se NPs) frequently undergo aggregation in aqueous environments, forming gray-black elemental selenium deposits. This process subsequently diminishes their bioactivity and bioavailability, thereby constraining their practical applications in biomedicine and related fields [9]. Consequently, identifying effective stabilizing agents and dispersing agents emerges as a critical strategy to enhance the bioavailability and biological activity of Se NPs.

To tackle the mentioned challenges, diverse biopolymers such as polysaccharides, proteins, and polyphenols have been utilized in recent years as stabilizing and dispersing agents for the surface modification of Se NPs, aiming to enhance their colloidal stability and functional performance [10]. The hydroxyl and amino functional groups present in these biomacromolecules engage in hydrogen bonding, hydrophobic interactions, or electrostatic interactions to lower the surface energy of Se NPs, consequently promoting their effective dispersion and colloidal stability in solution [11]. Among the array of stabilizing agents, polysaccharides stand out as optimal candidates for fabricating uniformly dispersed and stable selenium nanoparticles (Se NPs) in aqueous solutions. This superiority stems from their excellent water solubility, intricately branched molecular architecture, high specific surface area, and rich presence of hydroxyl groups, which collectively contribute to enhanced colloidal stability and controlled nanoparticle synthesis [12]. Furthermore, polysaccharides demonstrate a spectrum of advantageous biological activities, encompassing antioxidant, anti-inflammatory, antidiabetic, anticancer, and immunomodulatory properties. The advantageous characteristics of polysaccharides not only enable them to effectively enhance the stability of Se NPs, but also foster the synergistic interplay between polysaccharides and Se NPs [13]. Therefore, currently, polysaccharides have been widely used as modifiers to prepare stable Se NPs. For example, Lin et al. [14] synthesized Se NPs with diameters of 70 nm and 35 nm using green tea and Pu-Erh tea polysaccharides as stabilizers and dispersants. Subhash and colleagues [15] successfully prepared selenium nanoparticles (MPS-NPs) with a mean particle size of 89.2 nm utilizing date seed-derived polysaccharides, demonstrating remarkable stability as evidenced by sustained colloidal integrity over a 42-day storage period.

*Poria cocos*, as an edible fungus that grows on the roots of pine species, is widely distributed in China, Japan, India and other Asian countries. *Poria cocos* has been incorporated into daily diets since ancient times; in Chinese folk traditions, it is commonly prepared as traditional snacks like *Poria cocos* cake or *Poria cocos* porridge, or simmered with ingredients such as Chinese yam and lotus seeds to create nourishing soups [16]. It serves both as a staple food supplement and possesses health benefits including promoting diuresis and draining dampness. *Poria cocos* is also a medicinal plant with a therapeutic history of thousands of years, which was first recorded in the “Shennong Bencao Jing” [17]. With its neutral nature, sweet taste, and affinity for the heart, lung, spleen, and kidney meridians, *Poria cocos* has become a core herb for promoting diuresis, draining dampness, strengthening the spleen, and calming the heart [18]. It is widely used in classic formulas such as the Four Gentlemen Decoction and Six Flavor Rehmannia Pill. As one of the main components of *Poria cocos*, *Poria cocos* polysaccharides (PCP) have many beneficial biological activities, including immunomodulatory effects, anticancer properties, antioxidant activity, anti-inflammatory potential, as well as diuretic and sedative effects [19]. Considering the above beneficial values, we hypothesized that PCP may not only be an ideal stabilizer of Se NPs, but also significantly enhance the bioactivity of Se NPs to expand its application in selenium dietary supplements.

Based on the above background, in our work, PCP-functionalized selenium nanoparticles (designated as PCP-Se NPs) were fabricated through a chemical reduction strategy employing selenite and ascorbic acid as reactants, with PCP serving dual roles as both a stabilizing and dispersing agent. The structures of the obtained PCP-Se NPs were characterized using transmission electron microscopy, atomic force microscopy, Zetasizer Nano ZSE, energy dispersive spectrometer, X-ray photoelectron spectroscopy, infrared spectroscopy, ultraviolet spectroscopy and X-ray diffraction. This research systematically evaluated the gastrointestinal digestion behavior, colloidal stability, and in vitro bioactivities—including antioxidant and anti-inflammatory properties—of PCP-Se NPs. The findings offer compelling evidence for optimizing the stability of selenium nanomaterials while advancing the rational design of next-generation selenium-enriched nutraceuticals with enhanced biofunctionality.

## 2. Materials and Methods

### 2.1. Materials

*Poria cocos* polysaccharides were supplied by Beijing Solarbio Technology Co., Ltd. (Beijing, China), while sodium selenite (Na_2_SeO_3_) was acquired from Sigma Aldrich (St. Louis, MO, USA). Vitamin C (Vc) was sourced from Shanghai Aladdin Biochemical Technology Co., Ltd. (Shanghai, China). Simulated gastric fluid (SGF, pH 1.5) and simulated intestine fluid (SIF, pH 7.0) were obtained from Beijing Reagan Biotechnology Co., Ltd. (Beijing, China). 1,1-Diphenyl-2-picrylhydrazyl radical 2,2-Diphenyl-1-(2,4,6-trinitrophenyl) hydrazyl (DPPH) and 2, 2′-azino-bis(3-ethylbenzothiazoline-6-sulfonic acid) (ABTS) were purchased from Shanghai Macklin Biochemical Technology Co., Ltd. (Shanghai, China). Mouse monocyte macrophage RAW264.7 (database name: cellosaurus; Login number: CVCL_0493) was provided to Changsha Aibiwei Biotechnology Co., Ltd. (Changsha, China).

### 2.2. Synthesis of PCP-Modified Selenium Nanoparticles

Distilled water or PCP solution was thoroughly blended with 10 mM Na_2_SeO_3_ (2 mL) via gentle agitation. A 40 mM ascorbic acid (Vc) solution (2 mL) was then gradually added dropwise to the mixture, which was allowed to react for 24 h at ambient temperature. Following the reaction period, the mixture underwent 24 h dialysis (molecular weight cut-off: 500 Da) and subsequent freeze-drying to yield Se NPs or PCP-functionalized Se NPs (PCP-Se NPs), respectively.

### 2.3. Structural Characterization of PCP-Se NPs

Visual documentation of the synthesis pathways for both unmodified Se NPs and PCP-Se NPs was systematically captured. High-resolution morphological characterization was performed using transmission electron microscopy (TEM, JEOL Instruments, Tokyo, Japan) and atomic force microscopy (AFM, Hitachi High-Tech Corporation, Tokyo, Japan). The physicochemical properties of both Se NPs and PCP-Se NPs were comprehensively characterized: particle size distribution, polydispersity index (PDI), and surface charge (zeta potential) were quantified using a Zetasizer Nano ZSE system (Malvern Instruments Limited, Malvern, UK). Selenium content was determined via inductively coupled plasma optical emission spectrometry (ICP-OES) on an Optima 8000 platform (PerkinElmer, Waltham, MA, USA). Elemental composition and spatial distribution were analyzed through energy-dispersive X-ray spectroscopy (EDS) utilizing Oxford Instruments (Waltham, MA, USA) equipment, with specific quantification of elemental content in PCP-Se NPs to confirm successful functionalization. Surface chemical composition and elemental oxidation states of PCP-Se NPs were investigated through X-ray photoelectron spectroscopy (XPS) analysis, employing the Thermo Scientific K-Alpha system (Waltham, MA, USA). Molecular structural features and chemical bonding patterns of PCP, Se NPs and PCP-Se NPs were examined through Fourier-transform infrared (FT-IR) spectroscopy and ultraviolet-visible (UV-Vis) spectroscopy, both conducted using PerkinElmer instrumentation (Waltham, MA, USA). Crystal lattice structures were characterized via X-ray diffraction (XRD) analysis performed on a Shimadzu system (Kyoto, Japan).

### 2.4. In Vitro Simulated Digestion Evaluation of PCP-Se NPs

The digestive stability of PCP-Se NPs under simulated gastrointestinal conditions was evaluated based on the experimental protocol established by Liu and colleagues [20]. An aliquot of both Se NPs and PCP-Se NPs dispersions (10 mL) was combined with 10 mL of simulated gastric fluid (SGF, composed of hydrochloric acid (0.1 M), pepsin (3.2 g/L), etc., pH 1.2) and subjected to controlled agitation at 37 °C using a shaker set to 120 rpm for a duration of 120 min. Subsequently, the samples were reacted in a boiling water bath at 100 °C for 10 min to inactivate the enzyme. The above gastro-digested samples were mixed with 10 mL of simulated intestinal fluid (SIF, composed of phosphates (6.8 g/L) and trypsin (10 g/L), etc., pH 6.8) and placed in an oscillator (120 rpm, 37 °C) for 240 min. Finally, the samples were boiled at 100 °C to inactivate the enzyme. Under simulated gastrointestinal digestion conditions, 2 mL aliquots of digestive samples were systematically collected at defined time intervals (SGF: 10, 20, 30, 60, 120 min; SIF: 150, 180, 240, 300, 360 min) and immediately replaced with equivalent volumes of fresh simulated gastrointestinal fluid to preserve volume consistency throughout the experiment.

### 2.5. Measurement of Se Release Rate

The kinetics of selenium (Se) release were assessed using the experimental procedure developed by Xiao and collaborators [21]. Precisely measured 2 mL aliquots of gastric and intestinal digestates were transferred into conical flasks, followed by the addition of 10 mL of a 9:1 nitric acid-perchloric acid solution and several glass beads. The flasks were then sealed with watch glasses and left to undergo acid digestion under ambient conditions for an extended overnight period. After digestion, the above samples were heated on a hot plate and nitric acid was added promptly. When the sample solution became colorless and accompanied by white smoke, the sample solution was further heated to a remaining volume of about 2 mL. Following the cooling phase, 5 mL of 6 mol/L hydrochloric acid solution was carefully added to the sample mixture and subjected to controlled heating. The heating process continued until the solution achieved a completely colorless appearance, simultaneously accompanied by the characteristic emission of white smoke. After allowing the sample solution to cool, it was carefully transferred to a 10 mL volumetric flask. A volume of 2.5 mL of potassium ferricyanide solution (100 g/L) was then introduced, followed by dilution to the 10 mL calibration mark using deionized water. The release rate of Se from gastric and intestinal digestive fluids at different time points was determined by ICP.

### 2.6. Stability Evaluation of PCP-Se NPs

#### 2.6.1. Storage Stability

The stability of PCP-Se NPs was assessed after storage for 0, 10, 20, and 30 days. Specifically, PCP-Se NPs (1 mg/mL) were prepared. According to the method of Huang et al. [22] with minor modifications, 3 μL of sample was dispensed onto a carbon-coated copper grid and left at room temperature for 2 min. Excess liquid was removed with filter paper, followed by application of 2% phosphotungstic acid negative stain for 30 s, then blotted dry and dried. The changes in microstructure of PCP-Se NPs were measured by transmission electron microscopy. Additionally, following the method of Zou et al. [23], the particle size distribution (nm), particle size (nm), and polydispersity index (PDI, dimensionless) of PCP-Se-NPs at different storage times were measured using a Zetasizer Nano ZSE. And the changes in dispersion of PCP-Se NPs were recorded at different storage times by photographing the samples.

#### 2.6.2. Salt Ion Stability

The changes in microstructure, particle size distribution, particle size, and polydispersity index were measured at different salt ion concentrations (0 M–0.2 M) using transmission electron microscopy and Zetasizer Nano ZSE. The changes in sample dispersion were also observed.

### 2.7. Determination of Antioxidant Activity of PCP-Se NPs

The assessment of the free radical scavenging capacity of PCP-Se NPs was conducted using a modified version of the experimental protocol established by Song and co-researchers [24]. First, the different concentrations of PCP-Se NPs dispersions (12.5, 25, 50, 100, 200, 400 μg/mL, 0.1 mL) were mixed with an equal volume of DPPH solution and reacted for 30 min at room temperature in the dark. The absorbance at 517 nm was quantified for the reaction mixtures using a high-precision automated microplate analyzer (TECAN Infinite M200, Männedorf, Switzerland).

ABTS reserve solution (7 mM) was added to potassium persulfate solution (2.5 mM) and reacted at room temperature for 16 h protected from light. A volume of 0.1 mL from a series of PCP-Se NPs dispersions at concentrations of 12.5, 25, 50, 100, 200, and 400 μg/mL was combined with a standardized ABTS working solution diluted to achieve an initial absorbance of 0.70 ± 0.02 at 517 nm. The mixture was then incubated under ambient laboratory conditions (25 ± 2 °C) for a precise duration of 30 min. Final spectral absorption measurements at 734 nm were performed on the reaction mixtures using an automated multichannel microplate spectrophotometer. Ethanol served as the negative control for baseline correction.

### 2.8. Assessment of Cellular Uptake Behavior of PCP-Se NPs

To investigate the uptake behavior of cells toward PCP-Se-NPs, PCP-Se NPs were fluorescently labeled with fluorescein isothiocyanate (FITC) according to the method of Yang et al. [25] with minor modifications. Initially, Se NPs and PCP-Se NPs were suspended in phosphate-buffered saline (PBS). A FITC solution at 1 mg/mL concentration was then introduced to the dispersion, followed by gentle agitation under light-protected conditions at 4 °C for 12 h to facilitate covalent conjugation. The resulting fluorescently labeled nanoparticle suspensions were subsequently processed by centrifugation at 10,000 rpm for 15 min and subjected to three successive PBS washing cycles to remove unbound FITC.

The supernatant after centrifugation was diluted with PBS and measured for fluorescence intensity (F_Sample_) using a fluorometer (excitation wavelength 495 nm, emission wavelength 525 nm). Simultaneously, the initial FITC solution (1 mg/mL) was diluted proportionally, and its fluorescence intensity (F_Total_) was measured. Labeling efficiency (LE) was calculated using the following formula:

(1) $$LE(\%)=\left[1-F_{Sample}/F_{Total}\right]\times 100\%$$

After measurement, the FITC labeling efficiencies of Se NPs and PCP-Se NPs were 62.25 ± 3.52% and 50.54 ± 2.16%, respectively.

The macrophage RAW264.7 cells were seeded into 6-well culture plates at a density of 1 × 10^6^ cells per well and maintained under standard incubation conditions for 24 h. Subsequently, the adherent cells were co-incubated with FITC-Se NPs at 20 μg/mL concentration and FITC-PCP-Se NPs at an equivalent selenium concentration of 20 μg/mL for a duration of 6 h. Post-incubation, the culture supernatant was aspirated, and the adherent cells underwent three consecutive PBS rinses. The fixed-cell protocol was then implemented using 4% paraformaldehyde solution for 15 min immersion to maintain morphological integrity. Subsequent nuclear visualization was achieved through 10 min DAPI counterstaining. Cellular fluorescence localization was visualized and documented through high-resolution fluorescence inverted microscopy (Nikon Corporation, Tokyo, Japan). Quantitative evaluation of intracellular fluorescence signal intensity was performed using Image J 1.52a software to objectively determine nanoparticle uptake efficiency.

### 2.9. Determination of Endocytosis Pathway of PCP-Se NPs

The endocytosis pathways of PCP-Se NPs were determined using the method by Chen et al. [26] with slight modifications. In short, the RAW264.7 cells were plated at 1 × 10^6^ cells/well density in 6-well tissue culture plates and allowed to adhere under standard humidified incubation (37 °C, 5% CO_2_) for 24 h. Prior to nanoparticle exposure, cellular monolayers underwent preconditioning with three distinct endocytic pathway inhibitors—chlorpromazine hydrochloride (CPZ), filipin III (FLI), and cytochalasin D (CyD)—each at 5 μg/mL concentration, through 30 min co-incubation under light-protected conditions. Following two successive PBS washes to eliminate inhibitor residues, the cells were exposed to both Se NPs and PCP-Se NPs for 6 h. Post-treatment cellular lysates were subjected to acid digestion protocols to solubilize intracellular selenium, followed by quantitative analysis of total selenium content using ICP-OES. The control experimental arm consisted of cell populations exposed exclusively to Se NPs and PCP-Se NPs under identical incubation parameters but without prior inhibitor pretreatment.

### 2.10. Evaluation of Lysosomal Escape Behavior

The lysosomal escape behavior of PCP-Se NPs was determined using the method of Tang et al. [27] with slight modifications. Briefly, the RAW264.7 cells were seeded into 6-well tissue culture plates at a density of 1 × 10^6^ cells per well and maintained under standard incubation conditions (37 °C, 5% CO_2_, humidified atmosphere) for 24 h. Subsequently, the adherent cells were exposed to FITC-PCP-Se NPs at an equivalent selenium concentration of 20 μg/mL for three distinct time intervals (2 h, 6 h and 12 h). Next, cells were incubated with culture medium containing Lyso-Tracker Red (50 nM, Beyotime Biotechnology Co., Ltd., Shanghai, China) for 30 min away from light, followed by PBS washing for 2 times. Adherent cells underwent immobilization via 15 min immersion in 4% paraformaldehyde solution to preserve morphological structures, followed by dual PBS rinsing cycles to eliminate residual fixative. Nuclear compartment visualization was achieved through 10 min incubation with DAPI counterstain, succeeded by two additional PBS washes. Processed specimens were then systematically examined using fluorescence inverted microscopy (Nikon Instruments Inc., Tokyo, Japan). The degree of co-localization between FITC-PCP-Se NPs and lysosomal compartments was quantitatively assessed through Pearson correlation coefficient computation, utilizing the colocalization analysis module within Image J software.

### 2.11. Evaluation of In Vitro Anti-Inflammatory Activity

The in vitro anti-inflammatory activity of nanoparticle samples was evaluated using the method by Zhang et al. [28] with minor modifications. Briefly, the RAW264.7 cells were seeded into six-well culture plates at a density of 1 × 10^6^ cells per well and incubated under standard physiological conditions (37 °C, 5% CO_2_, humidified atmosphere) for a 24 h period. Following initial culture establishment, the cells were sequentially exposed to three experimental conditions: (1) PCP at concentrations matching its equivalent in PCP-Se NPs, (2) Se NPs at 20 μg/mL, and (3) PCP-Se NPs at an equivalent selenium dose of 20 μg/mL. Each treatment group underwent 24 h incubation under standard tissue culture conditions (37 °C, 5% CO_2_). Subsequent to nanoparticle exposure, cultures were further maintained for an additional 24 h period in complete medium supplemented with lipopolysaccharide (LPS) at 1 μg/mL. Finally, the inflammation level in the cells was determined using myeloperoxidase (MPO), nitric oxide (NO), inducible nitric oxide synthase (iNOS) kits and Elisa kits (TNF-α, IL-1β and IL-10).

### 2.12. Effect of PCP-Se NPs on Oxidative Stress Levels

The effects of PCP-Se NPs on intracellular oxidative stress levels were analyzed using the method of Dong et al. [29] with minor modifications. In short, RAW264.7 cells were inoculated in 6-well plates (1 × 10^6^/well) and incubated for 24 h. Cells were co-incubated with PCP, Se NPs and PCP-Se NPs for 24 h, followed by exposure to LPS (1 μg/mL) for 24 h. ROS levels in the cells were detected using the DCFH-DA probe and observed by fluorescence inverted microscope. In addition, the biochemical profiling was conducted to quantify cellular oxidative stress parameters using standardized kits for catalase (CAT), total superoxide dismutase (SOD), reduced glutathione (GSH) and malondialdehyde (MDA).

### 2.13. Effect of PCP-Se NPs on Mitochondrial Membrane Potential

The effect of PCP-Se NPs on intracellular mitochondrial membrane potential homeostasis was determined using the method of Zhang et al. [30] with minor modifications. Briefly, RAW264.7 cells were established in six-well culture plates at a standardized seeding density of 1 × 10^6^ cells per well and cultured under physiological conditions (37 °C, 5% CO_2_ humidified incubator) for 24 h. Following this culture phase, experimental groups were systematically exposed to PCP, Se NPs, and PCP-Se NPs for 24 h. The cell cultures were further stimulated with lipopolysaccharide (LPS) at 1 μg/mL for an additional 24 h period. Cellular specimens were processed according to the standardized protocol of the mitochondrial membrane potential detection kit. Alterations in mitochondrial membrane potential were subsequently visualized and analyzed using fluorescence inverted microscopy.

### 2.14. Statistical Analysis

Data were processed using GraphPad Prism 10.1.2 (GraphPad Software, San Diego, CA, USA). All data were analyzed using one-way analysis of variance (ANOVA) and Tukey’s post hoc test. The significance level was * *p* < 0.05, ** *p* < 0.01, *** *p* < 0.001 and **** *p* < 0.0001. All experiments and analyses were in triplicate, and data were presented as mean ± standard deviation.

## 3. Results

### 3.1. Dispersion Evaluation of PCP-Se NPs

**Figure 1:**
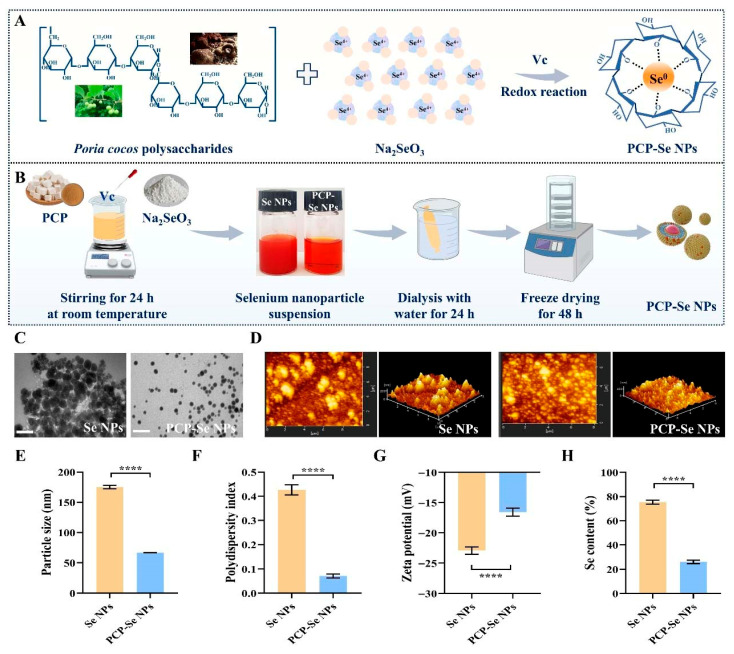
Physicochemical property analysis of Se NPs and PCP-Se NPs. (**A**) Schematic diagram of PCP-Se NPs synthesis. (**B**) The synthesis process of PCP-Se NPs. (**C**) Transmission electron microscope images and (**D**) atomic force microscope images of Se NPs and PCP-Se NPs. Scale bar = 200 nm. (**E**) The particle size, (**F**) polydispersity index and (**G**) zeta potential of Se NPs and PCP-Se NPs. (**H**) Se content determination of Se NPs and PCP-Se NPs. **** *p* < 0.0001.

Se NPs and PCP-Se NPs were synthesized through an oxidation-reduction system of Na_2_SeO_3_ and Vc (Figure 1A). Optical micrographs capturing the dispersion characteristics of both Se NPs and PCP-Se NPs were systematically acquired following a 24 h reaction period (Figure 1B). Both nanomaterial formulations presented uniform orange-red coloration in their aqueous suspensions, reflecting consistent colloidal stability and characteristic light absorption properties inherent to selenium-based nanomaterials. However, significant precipitation was observed in the unmodified Se NPs dispersion. As anticipated, PCP-Se NPs demonstrated a homogeneous and optically transparent dispersion state. This characteristic was plausibly linked to the profusion of hydroxyl functional groups within the PCP structure, which adsorbed onto the nanoparticle surfaces through hydrogen bonding or electrostatic interactions. Such surface modification effectively sterically hindered particle aggregation by creating an interfacial barrier that maintains colloidal stability [31]. Chen et al. [32] synthesized SFPS-modified selenium nanoparticles using *Sargassum fusiforme* polysaccharide (SFPS) as a modifier. The results revealed that SFPS significantly enhanced both the colloidal stability and dispersion homogeneity of selenium nanoparticles, as evidenced by quantitative particle size analysis and zeta potential measurements. Our study obtained consistent results.

### 3.2. Microscopic Morphology Analysis of PCP-Se NPs

The findings revealed that pristine Se NPs displayed pronounced aggregation phenomena, manifesting as the formation of substantial particulate conglomerates. This behavior was fundamentally attributed to the elevated surface energy characteristic of unmodified Se NPs, which induced spontaneous interparticle attraction and subsequent assembly into macroscopic clusters [33]. In contrast, PCP-Se NPs exhibited a monodisperse, uniformly distributed circular morphology with significantly diminished particle dimensions (Figure 1C). This structural optimization was likely attributable to the incorporation of PCP as a dispersing agent, which mitigated the inherent high surface energy of Se NPs through steric or electrostatic stabilization mechanisms. In addition, from the 2D and 3D images of atomic force microscopy, unmodified Se NPs exhibited pronounced aggregation phenomena accompanied by enlarged particle dimensions (Figure 1D). The maximum height was approximately 200 nm. Whereas the PCP-modified Se NPs featured a uniform distribution, and the maximum height of PCP-Se NPs was approximately 100 nm.

### 3.3. Particle Size, Polydispersity Index and Zeta Potential Analysis of PCP-Se NPs

The particle size of Se NPs was 175.25 ± 2.63 nm, and compared with Se NPs, the particle size of PCP-Se NPs was significantly reduced (66.64 ± 0.30 nm) (Figure 1E). It could also be observed that the particle size of PCP-Se NPs measured by Zetasizer Nano ZSE was smaller than that measured by transmission electron microscopy. This change was attributed to the shrinkage of the sample used for testing transmission electron microscopy during the drying process [34]. According to reports, the polydispersity index is a key indicator for evaluating the stability and dispersibility of solutions. Obviously, the polydispersity index of PCP-Se NPs (0.07 ± 0.006) was also much lower than that of Se NPs (0.43 ± 0.02) (Figure 1F). These findings reaffirmed the superior colloidal dispersion properties of PCP-Se NPs. According to previous studies, it was proved that the closer the zeta potential is to ±30 mV, the more stable the dispersed system is [35]. Quantitative analysis revealed zeta potential values of −22.93 ± 0.61 mV for unmodified Se NPs and −16.57 ± 0.68 mV for PCP-Se NPs (Figure 1G). The zeta potentials of Se NPs, *Paeonia lactiflora* polysaccharide (PLP) and PLP-Se NPs measured by Wang et al. [36] were −25.2 ± 0.45 mV, −19.9 ± 3.67 mV and −12.4 ± 0.31 mV, respectively. The results revealed that the absolute zeta potential magnitude of PLP-Se NPs was measurably lower than that of unmodified Se NPs. This reduction in absolute charge value, as explained by the authors, stems from the charge-shielding mechanism exerted by PLP moieties. Our results were consistent with previous reports. Of note, this may be an important reason for the enhanced stability of PCP-Se NPs. It has been reported that polysaccharides attenuate the absolute intensity of negative charges on Se NP surfaces through charge shielding effects, while maintaining particle dispersion Via steric hindrance from molecular chains and hydrogen bond networks [37]. Furthermore, electrostatic repulsion between polysaccharides and Se NPs is critical for stability. Polar groups in polysaccharide molecules form a dynamic charge equilibrium with the Se NPs surface. This reduces the absolute zeta potential value while simultaneously inhibiting particle aggregation through the synergistic action of electrostatic repulsion and steric stabilization.

### 3.4. Se Content and Elemental Distribution Analysis of PCP-Se NPs

**Figure 2:**
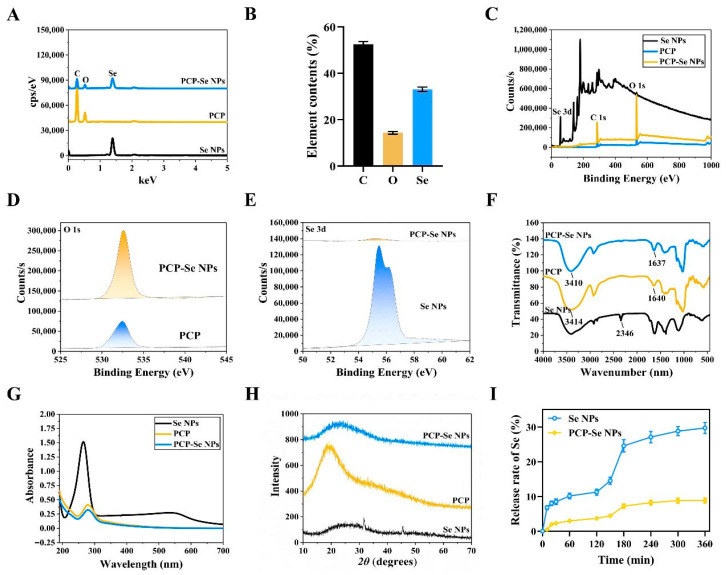
Chemical structure characterization of Se NPs and PCP-Se NPs. (**A**) Elemental distribution of PCP, Se NPs and PCP-Se NPs. (**B**) Elemental content analysis of PCP-Se NPs. (**C**) X-ray photoelectron spectra of PCP, Se NPs and PCP-Se NPs. (**D**) O 1s spectra of PCP and PCP-Se NPs. (**E**) Se 3d spectra of Se NPs and PCP-Se NPs. (**F**) Infrared spectra, (**G**) UV-visible spectra and (**H**) X-ray diffraction spectra analysis of PCP, Se NPs and PCP-Se NPs. (**I**) in vitro simulated digestion of Se NPs and PCP-Se NPs.

Quantitative elemental analysis confirmed that the selenium content in Se NPs and PCP-Se NPs was determined to be 75.34 ± 1.72% and 25.95 ± 1.38%, respectively (Figure 1H). Additionally, as shown in Figure 2A, Se NPs contained only Se elements, and PCP-Se NPs contained C, O, and Se elements. Elemental composition analysis confirmed that the presence of carbon (C) and oxygen (O) in PCP-Se NPs originated from the incorporation of PCP moieties, providing evidence for the successful construction of the functionalized nanomaterial system. Quantitative assessment of elemental distribution in PCP-Se NPs revealed carbon content at 52.58 ± 1.09%, oxygen at 14.31 ± 0.57%, and selenium at 33.11 ± 0.97% (Figure 2B).

### 3.5. Surface Chemical Composition of PCP-Se NPs

X-ray photoelectron spectroscopy analysis revealed distinct elemental signatures: in the PCP reference spectrum, an O 1s peak at 532.4 eV confirmed oxygen incorporation, while unmodified Se NPs exhibited characteristic Se 3d doublets at 55.5 eV and 56.2 eV, corresponding to selenium’s oxidation states (Figure 2C). For PCP-Se NPs, spectroscopic examination identified shifted binding energies: O 1s at 532.6 eV and Se 3d at 55.2 eV (Figure 2D,E). These spectral shifts provided evidence for Se-O bond formation, a key structural feature explaining the reduced nanoparticle size and enhanced colloidal stability observed in PCP-Se NPs.

### 3.6. Infrared Spectra Analysis

Spectroscopic examination of the infrared profile in Figure 2F identified prominent absorption bands: the sharp peak at 3410 cm^−1^ corresponded to hydroxyl (-OH) stretching vibrations, the signal at 2927 cm^−1^ aligned with methylene (-CH) stretching modes, and the feature at 1640 cm^−1^ was indicative of carbonyl (C=O) bending deformations. It could be clearly observed that PCP and PCP-Se NPs have similar infrared spectra, and no new absorption peaks appeared in the infrared spectra of PCP-Se NPs, suggesting that no new chemical bond was formed between PCP and Se NPs. Notably, the signal of -OH in PCP-Se NPs was shifted to 3410 cm^−1^ compared with that of PCP, indicating that hydrogen bonding interactions occurred between the -OH groups of PCP and Se NPs. The characteristic peak of C=O was blue shifted to 1637 cm^−1^, indicating the bonding between the C=O group in PCP and Se NPs. Similar studies showed that *Morchella sextelata* polysaccharides [38], *Cyperus esculentus* polysaccharides [39] and polysaccharides extracted from *Citrus limon* (L.) Burm. f. (Rutaceae) [40] had O-H groups that bound to Se NPs. Building on these findings, we proposed that the suppression mechanism of PCP in mitigating aggregation and precipitation phenomena of Se NPs may arise from the molecular interactions between Se NPs and hydroxyl functional groups (-OH).

### 3.7. UV Visible Spectral Analysis

Throughout the preparetion process of both Se NPs and PCP-Se NPs, the dispersion hue transitioned from transparent to orange/reddish-orange, which correlated with the presence of a distinct absorption maximum within the ultraviolet-visible spectrum, specifically spanning the 200–400 nm wavelength range. As could be seen from Figure 2G, Se NPs showed a maximum absorption peak around 265 nm. Previous studies have shown that this phenomenon is related to the excitation of plasma vibrations on the surface of Se NPs [41]. In contrast to Se NPs, the UV-Vis absorption profile of PCP-Se NPs closely mirrored that of PCP, exhibiting no trace of the characteristic absorption peaks inherent to Se NPs, which further validates the molecular-level integration of PCP moieties into the nanoconjugate architecture.

### 3.8. X-Ray Diffraction Spectroscopy Analysis

The crystal structures of Se NPs, PCP, and PCP-Se NPs were recorded. Sharp characteristic diffraction peaks appeared in Se NPs at around 31° and 45°, indicating the presence of crystalline Se in the system (Figure 2H) [42]. However, these typical peaks disappeared in the X-ray diffraction peaks of PCP-Se NPs. In contrast, a single broad diffraction peak was observed around 2θ = 20°, indicating that the PCP-Se NPs existed in an amorphous structure of non-crystalline nature. This change may be related to the modification of PCP on the surface of Se NPs further disrupting the crystallinity of Se, and a similar phenomenon has been observed in some reports [43].

### 3.9. In Vitro Analysis of Simulated Digestive Behavior

The digestion release kinetics of selenium, as visualized in Figure 2I, revealed distinct patterns between nanoparticle formulations. Following simulated gastric digestion, both Se NPs and PCP-Se NPs exhibited limited initial release of selenium. Quantitative analysis at the 120 min mark demonstrated that Se NPs released 11.24 ± 0.89% of their selenium content into gastric fluid, whereas PCP-Se NPs showed a markedly reduced release rate of 3.69 ± 0.35%. During the intestinal digestion phase, a temporal escalation in selenium release rates was observed. Specifically, Se NPs exhibited a pronounced surge in selenium liberation, a phenomenon directly correlated with trypsin-mediated degradation within intestinal fluids. Enzymatic hydrolysis by trypsin compromised the structural integrity of Se NPs, triggering accelerated dissolution and consequent rapid release of elemental selenium. Interestingly, the release rate of Se NPs was more significant than that of PCP-Se NPs throughout the simulated digestion. At the 360 min interval of simulated digestion, quantitative analysis revealed that unmodified Se NPs exhibited a selenium release rate of 29.72 ± 1.63%, while their PCP-functionalized counterparts (PCP-Se NPs) showed a substantially lower release of 8.83 ± 0.73%. Notably, through all stages of the simulated gastrointestinal process, Se NPs consistently demonstrated markedly higher selenium liberation compared to PCP-Se NPs. Our results were in agreement with those reported by Zhang et al. [44]. This may be due to the fact that polysaccharide-modified Se NPs have a better stabilized structure in the gastrointestinal environment. It could be found that PCP-Se NPs exhibited superior stability in simulating the gastrointestinal tract digestion process compared with Se NPs, which has potential application for developing oral Se formulations.

### 3.10. Evaluation of Storage Stability and Salt Ion Stability of PCP-Se NPs

**Figure 3:**
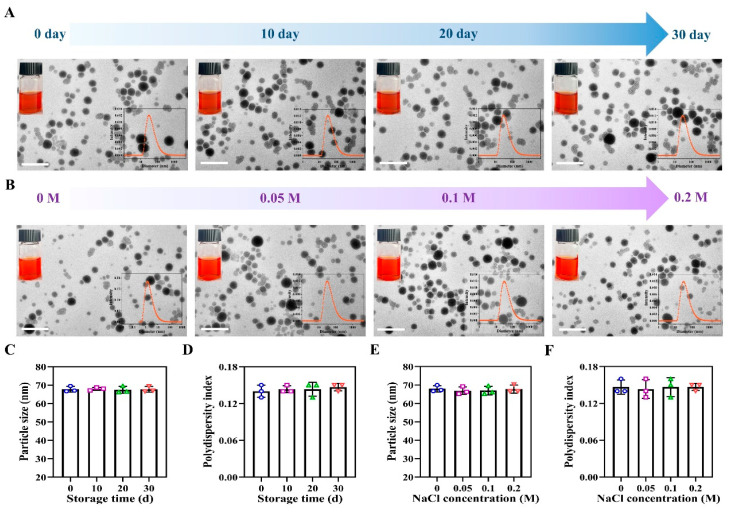
Stability analysis of Se NPs and PCP-Se NPs. (**A**) Storage stability of PCP-Se NPs. Illustration: sample images and particle size distribution images of PCP-Se NPs stored for 0, 10, 20, and 30 days. Scale bar = 200 nm. (**B**) Salt ion stability of PCP-Se NPs. Illustration: Sample images and particle size distribution of PCP-Se NPs at salt ion concentrations of 0 M, 0.05 M, 0.1 M, and 0.2 M. Scale bar = 200 nm. Changes in (**C**) particle size and (**D**) polydispersity index of PCP-Se NPs stored for different times. Changes in (**E**) particle size and (**F**) polydispersity index of PCP-Se NPs at different salt ion concentrations.

As shown in Figure 3A, the dispersions of PCP-Se NPs were well dispersed at different times of storage without aggregation and precipitation. The PCP-Se NPs showed a uniform spherical shape throughout the experimental period, and the particle size distribution, particle size and polydispersity index did not change significantly (Figure 3C,D). In addition, the dispersion, micro-morphology, particle size distribution, particle size and polydispersity index of PCP-Se NPs did not change significantly at different salt ion concentrations (Figure 3B,E,F). The experimental findings collectively demonstrated that PCP-Se NPs displayed remarkable stability characteristics, establishing a robust foundation for advancing subsequent research endeavors.

### 3.11. Evaluation of In Vitro Antioxidant Activity

**Figure 4:**
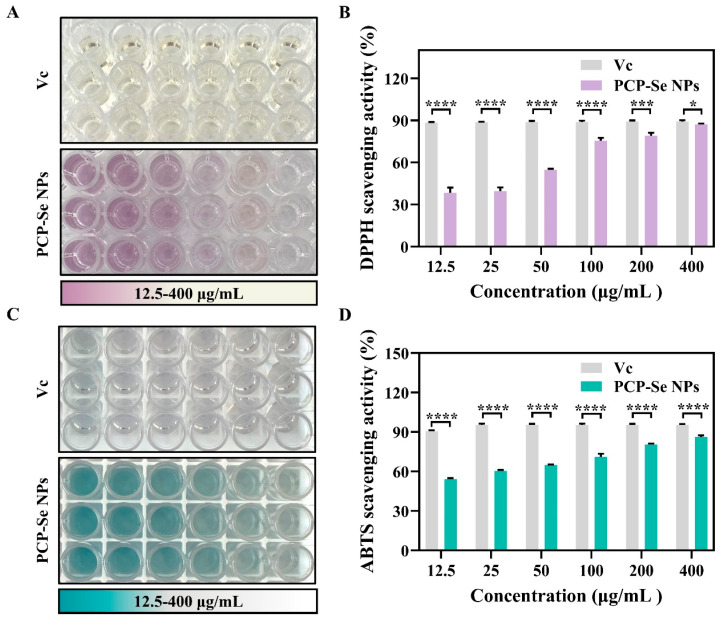
In Vitro antioxidant activity evaluation of PCP-Se NPs. (**A**) Color change and (**B**) DPPH radical scavenging activity of different concentrations of PCP-Se NPs after reacting with DPPH radicals. (**C**) Color change and (**D**) ABTS radical scavenging activity of different concentrations of PCP-Se NPs after reacting with ABTS radicals. * *p* < 0.05, *** *p* < 0.001 and **** *p* < 0.0001.

DPPH is considered a very stable lipophilic free radical and can be used to evaluate the antioxidant activity of substances. As shown in Figure 4A,B, the DPPH scavenging rate showed an increasing trend as the concentration of PCP-Se NPs increased, and the color of the samples changed from purple to lavender. When the concentration was 400 μg/mL, the DPPH scavenging rate reached 87.18 ± 0.64%, indicating that the PCP-Se NPs possessed strong DPPH scavenging activity. After calculation, the IC_50_ values for Vc and PCP-Se NPs in scavenging DPPH radicals were 7.06 μg/mL and 32.18 μg/mL, respectively.

In addition, the scavenging activity of ABTS radicals was determined. As shown in Figure 4C,D, the color of the samples gradually changed from cyan to light and even to colorless with the increase in sample concentration. Correspondingly, the scavenging rate of ABTS radicals also showed a concentration-dependent increase. The ABTS scavenging rate of PCP-Se NPs at 400 μg/mL was about 86.28 ± 1.17%. Notably, the IC_50_ value for PCP-Se NPs in scavenging ABTS radicals (9.94 μg/mL) was comparable to that of Vc (6.91 μg/mL). The above results demonstrated that PCP-Se NPs possessed potent antioxidant activity in vitro. According to recent reports, the smaller the particle, the larger the specific surface area, which can provide a large number of free radical reaction sites [45]. In this study, the modification of PCP significantly reduced the particle size of Se NPs compared with Se NPs, which may account for the excellent antioxidant activity of PCP-Se NPs.

### 3.12. MTT Result Analysis

MTT results showed that cells treated with Se NPs and PCP-Se NPs at a Se concentration of 20 μg/mL exhibited the highest cell viability (Figure S1). Based on this, subsequent cell experiments in this study were conducted using a Se concentration of 20 μg/mL.

### 3.13. Analysis of Cellular Uptake Capacity of PCP-Se NPs

**Figure 5:**
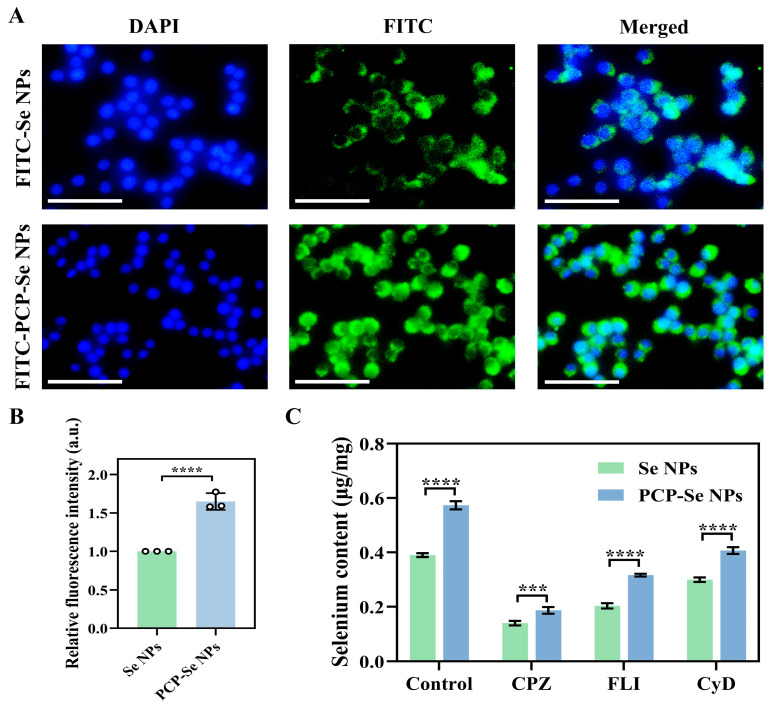
Cellular uptake evaluation of PCP-Se NPs. (**A**) Fluorescence images and (**B**) average fluorescence intensity of cellular uptake of FITC-PCP-Se NPs. Magnification: 10 times. Scale bar: 200 μm. (**C**) Analysis of endocytosis pathways of Se NPs and PCP-Se NPs. *** *p* < 0.001 and **** *p* < 0.0001.

The efficacy of cellular internalization for nanoparticles is pivotal to their therapeutic performance, necessitating a comparative investigation into the uptake ability of FITC-Se NPs and FITC-PCP-Se NPs. As illustrated in Figure 5A, intense green fluorescence signals localized adjacent to the nucleus were distinctly detectable, suggesting that both FITC-Se-NPs and FITC-PCP-Se-NPs were effectively internalized into the cytoplasmic compartment of RAW264.7 cells. Remarkably, the FITC-PCP-Se NP-treated cells exhibited a markedly greater intensity of intracellular green fluorescence compared to the unmodified FITC-Se NPs group, as evidenced by quantitative fluorescence microscopy analysis, demonstrating that pentachlorophenol surface functionalization significantly amplifies the cellular internalization efficiency of Se NPs. This phenomenon was further confirmed by the fluorescence quantification results in Figure 5B, evidenced by the significantly higher relative fluorescence intensity in the FITC-PCP-Se NPs group (1.65 ± 0.09) than in the unmodified FITC-Se NPs group. Studies have shown that nanoparticles with smaller particle sizes are more readily taken up by cells because they can cross the cell membrane more efficiently to be absorbed by the biological barrier [46]. For example, Zhang et al. [47] demonstrated that the smallest particle size (79 nm) of *Cordyceps sinensis* exopolysaccharide-selenium nanoparticles (EPS-SeNPs) had 1–2 times higher antitumor activity than particles of 124 nm. The results of Wang et al. [48] confirmed the stronger antiproliferative effect of Se NPs with smaller particle sizes, which the authors explained was mainly due to the more active electron donating and electron accepting ability of small-sized selenium nanoparticles with larger surface area. In this investigation, the average particle size of PCP-Se NPs was measured at 66.64 ± 0.30 nm, demonstrating a marked reduction compared to unmodified Se NPs (175.25 ± 2.63 nm) and falling well below previously published dimensions [49]. This size reduction is hypothesized to contribute to the enhanced antioxidant efficacy and anti-inflammatory properties observed in PCP-Se NPs, as nanoscale dimensions often correlate with improved cellular permeability and bioavailability. Notably, these findings establish a critical rationale for the utilization of FITC-labeled PCP-Se NPs as optimized selenium carriers in dietary supplementation strategies.

### 3.14. Evaluation of Endocytosis Pathway

The endocytosis pathways of nanoparticles mainly include clathrin-mediated endocytosis, caveolin-mediated endocytosis, and macropinocytosis. Consequently, the endocytic pathways of Se NPs and PCP-Se NPs in RAW264.7 macrophages were systematically investigated using three specific endocytosis inhibitors (CPZ, FLI, CyD). Findings from the experiments, as depicted in Figure 5C, revealed that control cells exhibited the highest intracellular selenium content. This observation indicated that both nanoparticle formulations efficiently traversed the cell membrane and underwent smooth internalization under baseline conditions, confirming that their cellular entry mechanisms remain unimpeded in the absence of inhibitors targeting endocytic processes. In comparison to untreated control cells, the introduction of endocytosis inhibitors (CPZ, FLI, CyD) led to a marked reduction in intracellular selenium levels for both nanoparticle formulations, indicating that Se NPs and PCP-Se NPs entered RAW264.7 macrophages. Strikingly, among the three endocytosis inhibitors tested, cells treated with CPZ exhibited the lowest intracellular selenium content, a finding that indicated clathrin-mediated endocytosis as the predominant pathway for cellular entry of both Se NPs and PCP-Se NPs, followed by the caveolin-mediated endocytosis and macropinocytosis. Notably, the Se content of PCP-Se NPs was significantly higher than that of Se NPs in all treatment groups. This observation underscored that PCP surface functionalization markedly augmented the cellular internalization efficacy of Se NPs. Zhang et al. [50] synthesized *Cordyceps sinensis* exopolysaccharide-selenium nanoparticles (EPS-SeNPs) and also obtained similar results to those of this study. The authors explained that the modification of fisetin sulfate altered the surface properties of Se NPs, such as charge, hydrophilicity, etc., which made it easier for them to interact with the cell membrane, thus promoting endocytosis. However, in this study, the specific molecular mechanisms (e.g., expression, localization, and interactions of relevant proteins) and influencing factors (particle size, charge, and hydrophilicity) of the endocytosis process of PCP-Se NPs in RAW264.7 cells have not yet been thoroughly investigated, and will need to be further explored in the future.

### 3.15. Analysis of Lysosomal Escape Behavior

**Figure 6:**
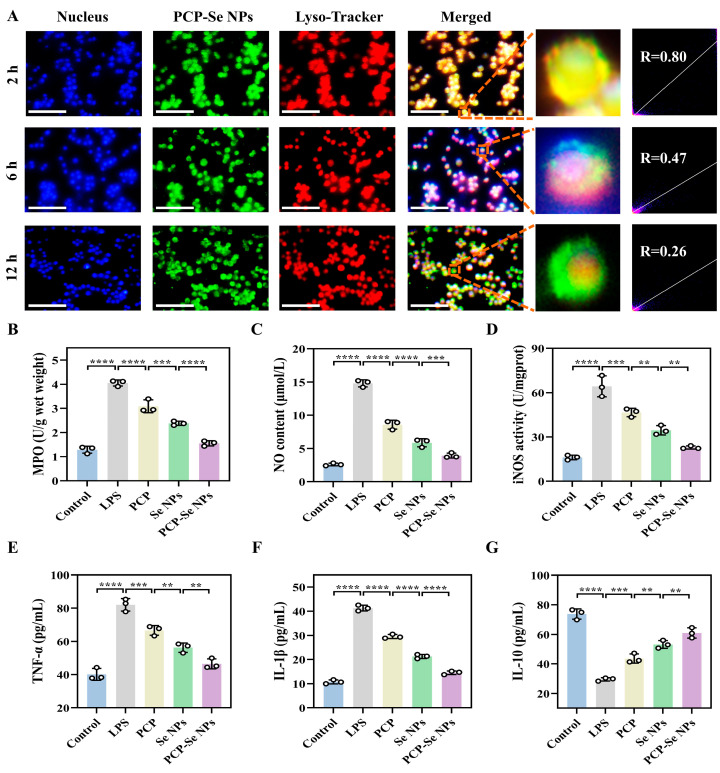
Evaluation of lysosomal escape ability and anti-inflammatory activity of PCP-Se NPs. (**A**) Fluorescence images of lysosomal escape of FITC-PCP-Se NPs in RAW264.7 cells at 2 h, 6 h, and 12 h, respectively. Magnification: 4 times. Scale bar: 100 μm. Changes of (**B**) MPO, (**C**) NO, (**D**) iNOS, (**E**) TNF-α, (**F**) IL-1β, and (**G**) IL-10 levels in RAW264.7 cells treated with PCP, Se NPs, and PCP-Se NPs, respectively. ** *p* < 0.01, *** *p* < 0.001 and **** *p* < 0.0001.

The lysosomal escape behavior of nanoparticles directly determines whether they can effectively avoid lysosomal degradation and deliver active ingredients to the cytoplasm to exert their effects, which is a key indicator for evaluating their practical application value. The experimental results showed that at 2 h, the red fluorescence of lysosomes basically overlapped with the green fluorescence of FITC-PCP-Se NPs, at which time the cells showed yellow fluorescence, and the Pearson’s correlation coefficient (R) was 0.80, which indicated that the FITC-PCP-Se NPs co-localized with lysosomes to a high degree and the NPs were effectively endocytosed (Figure 6A). At 6 h, the green fluorescence gradually separated from the red fluorescence, and the degree of co-localization of FITC-PCP-Se NPs with lysosomes decreased (R = 0.47), indicating that some of the FITC-PCP-Se NPs detached from lysosomes and entered the cytoplasm. At 12 h, the co-localization of FITC-PCP-Se NPs with lysosomes was significantly reduced (R = 0.26), indicating that FITC-PCP-Se NPs successfully escaped from lysosomes and were widely distributed in the cytoplasm. This result suggested that PCP-Se NPs could effectively escape from lysosomes and avoid being degraded, thus improving their bioavailability in the cytoplasm and providing an advantage for the subsequent drug to exert beneficial effects.

### 3.16. Effect of PCP-Se NPs on Inflammatory Factor Levels

To investigate the in vitro anti-inflammatory activity of PCP-Se NPs, the levels of inflammatory factors in RAW264.7 cells were first determined. As shown in Figure 6B–G, the levels of MPO, NO, iNOS, TNF-α, and IL-1β were significantly increased and the level of IL-10 was significantly decreased in the cells after treatment with LPS, suggesting the inflammatory damage of the cells. After treatment with PCP, Se NPs, and PCP-Se NPs, the levels of MPO, NO, iNOS, TNF-α, and IL-1β decreased, while the level of IL-10 significantly increased, which were 0.38 ± 0.013-fold, 0.26 ± 0.02-fold, 0.36 ± 0.02-fold, 0.57 ± 0.03-fold, 0.35 ± 0.02-fold, and 2.07 ± 0.16-fold that of the LPS group, respectively. These results indicated that they could effectively alleviate inflammation. In contrast, PCP-Se NPs showed the most prominent in vitro anti-inflammatory activity.

### 3.17. Effect of PCP-Se NPs on the Level of Oxidative Stress

**Figure 7:**
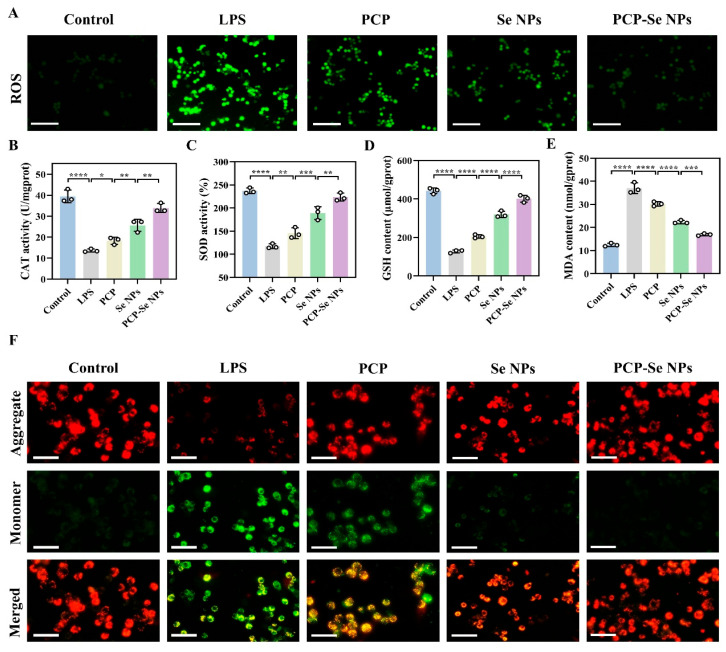
Effect of PCP-Se NPs on oxidative stress levels in RAW264.7 cells. (**A**) Fluorescence images of ROS in RAW264.7 cells treated with PCP, Se NPs and PCP-Se NPs. Magnification: 10 times. Scale bar: 200 μm. The changes in (**B**) CAT, (**C**) SOD, (**D**) GSH, and (**E**) MDA levels in RAW264.7 cells treated with PCP, Se NPs, and PCP-Se NPs. (**F**) Fluorescence images of mitochondrial membrane potential in RAW264.7 cells treated with PCP, Se NPs, and PCP-Se NPs, respectively. Magnification: 10 times. Scale bar: 200 μm. * *p* < 0.05, ** *p* < 0.01, *** *p* < 0.001 and **** *p* < 0.0001.

It is widely recognized that ROS serve as a critical biomarker for assessing cellular oxidative stress status. Overaccumulation of ROS induces oxidative stress conditions, which subsequently trigger oxidative stress-related cellular damage. Given these mechanistic links, intracellular ROS concentrations were systematically quantified to monitor oxidative stress levels and evaluate potential cytotoxic effects. Following LPS stimulation, intense green fluorescence was distinctly visualized, signifying robust overproduction of ROS and subsequent oxidative stress-mediated cellular damage, as corroborated by fluorescence microscopy imaging (Figure 7A). Interestingly, PCP-Se NPs significantly scavenged ROS from the cells to maintain the balance of oxidative stress levels, as evidenced by the attenuation of green fluorescence. The quantification of ROS relative fluorescence intensity strongly supported the above results (Figure S2). The cellular antioxidant capacity was additionally evaluated. As demonstrated in Figure 7B–E, exposure to PCP-Se NPs led to a significant elevation in key antioxidative biomarkers—including CAT, SOD, and GSH—while concomitantly reducing the concentration of MDA, a lipid peroxidation biomarker indicative of oxidative stress severity, which were 2.48 ± 0.02-fold, 1.91 ± 0.11-fold, 3.16 ± 0.28-fold, and 0.46 ± 0.03-fold that of the LPS group, respectively. This indicated that PCP-Se NPs treatment enhanced the antioxidant defense capacity of cells. These interesting findings highlighted the positive role of PCP-Se NPs in ameliorating oxidative stress. Studies have been reported to demonstrate the mitigating effects of Se NPs on inflammation and oxidative stress. For example, Ouyang et al. [51] demonstrated that selenium nanoparticle hydrogel microbeads significantly ameliorated ulcerative colitis and modulated intestinal immunity and microbiota through the TLR4-NF-κB pathway. Xiao et al. [52] demonstrated significant improvement in Metabolic dysfunction-associated fatty liver disease (MAFLD) by activating the Nrf2-mediated GSH-GPX4 pathway.

Notably, compared to unmodified Se NPs, PCP-Se NPs exhibited a more prominent alleviating effect on oxidative stress. Beyond the intrinsic effects of selenium nanoparticles, this phenomenon may also involve synergistic contributions from PCP—for example, PCP as a surface modifier could enhance nanoparticle biocompatibility, promote cellular uptake efficiency, or directly participate in radical scavenging through its chemical structure, thereby forming a complementary or enhanced antioxidant network with selenium. However, this study did not validate the necessity of PCP through blocking experiments (e.g., inhibiting PCP binding sites). Future work will systematically elucidate the specific action sites and synergistic mechanisms of PCP within antioxidant pathways using transcriptomics and protein interaction analysis, thereby more precisely clarifying the molecular basis of the PCP-Se-NPs antioxidant effect.

### 3.18. Depolarization Analysis of Mitochondrial Membrane Potential

Studies have shown that mitochondrial membrane potential is an important indicator of mitochondrial function and is closely related to inflammation and apoptosis. Based on this, changes in mitochondrial membrane potential in RAW264.7 cells were evaluated. Obviously, compared with the control group, red fluorescence was significantly weakened and green fluorescence was significantly enhanced in LPS group, suggesting that mitochondrial function was damaged and severe depolarization occurred (Figure 7F). Studies have reported that excessive accumulation of ROS leads to cellular oxidative stress, which in turn leads to mitochondrial dysfunction. This result was consistent with that of ROS. As expected, PCP-Se NPs had a significant role in significantly maintaining the balance of mitochondrial membrane potential as well as protecting the mitochondrial function, as evidenced by the strong red fluorescence and weak green fluorescence. In addition, the ratio of red/green fluorescence intensity for each group of fluorescence images was quantified and similar results were obtained (Figure S3). Wen et al. [53] found that selenium nanoparticles (GFP-GA-SeNPs) stabilized by *Grifola frondosa* polysaccharide and gallic acid conjugates could reduce ROS levels and restore MMP, thereby inhibiting apoptosis. The present study was consistent with the results reported above.

### 3.19. Future Prospects

This study systematically evaluated the stability and biological activity of PCP-Se NPs through synthesis and characterization, providing scientific basis for developing novel selenium supplements. However, existing research still has limitations, and future exploration can be deepened in the following four aspects: (1) Utilize proteomics and transcriptomics technologies to reveal key intracellular signaling pathways regulated by PCP-Se NPs (e.g., Nrf2/Keap1 antioxidant pathway, NF-κB inflammatory pathway), thereby clarifying their molecular mechanisms of action. For example, immunoprecipitation can validate whether PCP modification enhances binding efficiency with cell membrane receptors by altering the surface charge of selenium nanoparticles, thereby optimizing cellular uptake and lysosomal escape processes. (2) Conduct animal studies to validate the In Vivo efficacy and safety of PCP-Se NPs. Establish mouse oxidative stress models to evaluate their antioxidant activity and anti-inflammatory effects while monitoring hepatic and renal function indicators to ensure biocompatibility. (3) Investigate synergistic effects between PCP and Se NPs, and explore the potential for combined applications of PCP-Se NPs with other active ingredients (e.g., vitamin C, polyphenolic compounds). Utilize high-throughput screening to identify adjuvant components that enhance PCP-Se NP bioactivity, developing novel synergistic formulations to broaden their applications in nutritional supplementation and disease treatment. (4) Develop novel selenium supplements or drug delivery systems leveraging the superior properties of PCP-Se NPs. For instance, utilize microfluidic technology to prepare PCP-Se NPs composite microspheres with uniform particle size and controllable drug loading capacity. This enables targeted delivery and controlled sustained release, enhancing their clinical application value in fields such as tumor therapy and neurodegenerative disease intervention. In the future, we will continue monitoring cutting-edge research in PCP-Se NPs, integrating interdisciplinary approaches to advance their transition from laboratory studies to clinical applications, thereby contributing innovative strategies to the advancement of nanobiomedical science.

## 4. Conclusions

This study successfully developed stabilized *Poria cocos* polysaccharide modified Se NPs (PCP-Se NPs) through a simple redox system. These PCP-Se NPs exhibited enhanced stability, prominent in vitro antioxidant activity and anti-inflammatory activity. This study provides an alternative method for improving the stability of Se NPs and developing new selenium rich dietary supplements.

## Data Availability

The original contributions presented in the study are included in the article/Supplementary Materials. Further inquiries can be directed to the corresponding authors.

## Supplementary Materials

The following supporting information can be downloaded at: https://www.mdpi.com/article/10.3390/foods14203555/s1, Figure S1: The effects of different concentrations of Se NPs and PCP-Se NPs on cell viability of RAW264.7 cells.; Figure S2: The relative fluorescence intensity of ROS in RAW264.7 cells treated with PCP, Se NPs and PCP-Se NPs; Figure S3: Red/green fluorescence intensity ratio of mitochondrial membrane potential in RAW264.7 cells treated with PCP, Se NPs and PCP-Se NPs, respectively.

## References

[1] Zeng Y., Lyu S., Yang Q., Du Z., Liu X., Shang X., Xu M., Liu J., Zhang T. (2025). Preparation, physicochemical characterization, and immunomodulatory activity of ovalbumin peptide–selenium nanoparticles. Food Chem..

[2] Yang Z., Hu Y., Yue P., Li H., Wu Y., Hao X., Peng F. (2023). Structure, stability, antioxidant activity, and controlled-release of selenium nanoparticles decorated with lichenan from *Usnea longissimi*. Carbohydr. Polym..

[3] Raza A., Raza H., Singh A., Sharma A.K. (2022). Impact of selenium nanoparticles in the regulation of inflammation. Arch. Biochem. Biophys..

[4] Qi X., Tong L., Lian H., Chen Z., Yang L., Wu Y., Jin S., Guo D. (2025). Selenium nanoparticles modified with Ophiocordyceps gracilis polysaccharides: Enhancing stability, bioavailability, and anti-inflammatory efficacy. Food Res. Int..

[5] Cao M., Qian Y., Sun Z., Liu S., Zheng R., Zhao L., Chen G. (2025). Preparation, characterization, and stability of selenium nanoparticles decorated with Mori Fructus polysaccharide and its protective effects in bisphenol A-induced Sertoli cells. Int. J. Biol. Macromol..

[6] Li Y., Liu T., Zheng R., Lai J., Su J., Li J., Zhu B., Chen T. (2024). Translational selenium nanoparticles boost GPx1 activation to reverse HAdV-14 virus-induced oxidative damage. Bioact. Mater..

[7] Chen C., Ma J., Duan S., Xue M., Yang Z., Ma Z., Ji J., Ma Y., Qing G., Guo K. (2025). Mitigation of ischemia/reperfusion injury via selenium nanoparticles: Suppression of STAT1 to inhibit cardiomyocyte oxidative stress and inflammation. Biomaterials.

[8] Liu T., Pan S., Zhou Q., Yang Z., Zhang Z., Liu H., He L., Lan J., Hua Y., Chen T. (2025). Selenium nanoparticles restrain recurrence of cervical cancer in drug-free period by inhibiting the expression of ABC transporters. Nano Today.

[9] Sun K., Ma L., Hou J., Li Y., Jiang H., Liu W., Cao R., Zhang L., Guo Y. (2025). Physalis peruviana heteropolysaccharide-conjugated selenium nanoparticles: Preparation, characterization, and promising applications in cancer therapy. Int. J. Biol. Macromol..

[10] Jia B., Yang W., Li H., Chang G., Zhang X., Zhang N., Wang S., Wei J., Li X., Gao W. (2025). Ophiopogonis Radix fructan-selenium nanoparticles for dual amelioration of ulcerative colitis and anti-colon cancer. Int. J. Biol. Macromol..

[11] Zhang J., Ji T., Yang X., Liu G., Liang L., Liu X., Wen C., Ye Z., Wu M., Xu X. (2022). Properties of selenium nanoparticles stabilized by *Lycium barbarum* polysaccharide-protein conjugates obtained with subcritical water. Int. J. Biol. Macromol..

[12] Yu N., Chen H., Zongo A.W.S., Lu Y., Nie X., Meng X. (2023). Development of selenium nanoparticles stabilized by novel polysaccharides from *Stauntonia brachyanthera* pulp for anti-hepatoma cell proliferation. Food Biosci..

[13] Liu X., Jesus S.G., Kong Z., Fan N., Mi Y., Wang Q., Huang Y., Li L., Lu J., Fan B. (2025). Polysaccharide nano-selenium in the regulation of neuroinflammation: A review of mechanisms, functional potential, and activity evaluation. Carbohydr. Polym..

[14] Lin X., Mu J., Chen Z., Zhang Y., Ye X., Gao X., Chen W., Luo Y., Li B. (2023). Stabilization and functionalization of selenium nanoparticles mediated by green tea and Pu-Erh tea polysaccharides. Ind. Crops Prod..

[15] Subhash A., Bamigbade G., Abdin M., Jarusheh H., Abu-Jdayil B., Liu S.Q., Palmisano G., Ali A., Kamal-Eldin A., Ayyash M. (2025). Date seeds polysaccharides as novel capping agents for selenium nanoparticles: Synthesis, characterization, stability, biological activities, and gut microbiota modulation. Food Chem..

[16] Chen L., Zhao S., Chen Q., Luo P., Li X., Song Y., Pan S., Wu Q., Zhang Y., Shen X. (2025). *Poria cocos* polysaccharides ameliorate AOM/DSS-induced colorectal cancer in mice by remodeling intestinal microbiota composition and enhancing intestinal barrier function. Int. J. Biol. Macromol..

[17] Ng C.Y.J., Lai N.P.Y., Ng W.M., Siah K.T.H., Gan R.Y., Zhong L.L.D. (2024). Chemical structures, extraction and analysis technologies, and bioactivities of edible fungal polysaccharides from *Poria cocos*: An updated review. Int. J. Biol. Macromol..

[18] Yu D., Ge K., Chen N., Wang Y., Xu H. (2025). Water-soluble polysaccharides derived from Poria cocos protect against LPS-induced renal injury through inhibiting the NF-κB-NOX4 signaling pathway. Int. J. Biol. Macromol..

[19] Lv Y., Yang Y., Chen Y., Wang D., Lei Y., Pan M., Wang Z., Xiao W., Dai Y. (2024). Structural characterization and immunomodulatory activity of a water-soluble polysaccharide from *Poria cocos*. Int. J. Biol. Macromol..

[20] Liu G., Ji T., Pan J., Liu D., Liang L., Wen C., Liu X., Li Y., Zhang J., Xu X. (2024). Study on the digestion and absorption property of *Lycium barbarum* polysaccharide-protein stabilized selenium nanoparticles from the perspective of stability in vitro. LWT-Food Sci. Technol..

[21] Xiao Y., Huang Q., Zheng Z., Ma H. (2021). Selenium release kinetics and mechanism from *Cordyceps sinensis* exopolysaccharide-selenium composite nanoparticles in simulated gastrointestinal conditions. Food Chem..

[22] Huang J., Wang F., Zhang Y., You C., Li X. (2025). Development of self-assembled (+)-Nootkatone delivery system using Gliadin-Carboxymethyl chitosan composite nanoparticles: Fabrication, characterization, and pharmaceutical application. Int. J. Biol. Macromol..

[23] Zou M., Shang Y., Zhao Z., Lai Z., Zhou W., Liu T. (2025). Hyaluronic acid modified emodin polymeric nanoparticles for improved antibacterial activity and food preservation. Food Hydrocoll..

[24] Song H., Wang Q., He A., Li S., Guan X., Hu Y., Feng S. (2022). Antioxidant activity, storage stability and in vitro release of epigallocatechin-3-gallate (EGCG) encapsulated in hordein nanoparticles. Food Chem..

[25] Yang H., Zhu C., Yuan W., Wei X., Liu C., Huang J., Yuan M., Wu Y., Ling Q., Hoffmann P.R. (2021). Mannose-rich oligosaccharides-functionalized selenium nanoparticles mediates macrophage reprogramming and inflammation resolution in ulcerative colitis. Chem. Eng. J..

[26] Chen W., Li X., Cheng H., Zhan X., Xia W. (2022). Synthesis, characterization, and anticancer activity of protamine sulfate stabilized selenium nanoparticles. Food Res. Int..

[27] Tang X., Zhang J., Sun Y., Xu Z., Huang T., Liu X., Song Y., Zhang Y., Deng Y. (2025). Autonomic lysosomal escape via sialic acid modification enhances mRNA lipid nanoparticles to eradicate tumors and build humoral immune memory. J. Control Release.

[28] Zhang L., Zhao S., Wang J., Zhang J., Zheng T., Li J., Ma C., Liu J. (2025). Enzymatic degradation, structural characterization, and in vitro antioxidant, hypoglycemic, and anti-inflammatory activities of *Sanghuang vaninii* polysaccharides. Int. J. Biol. Macromol..

[29] Dong R., Wang Y., McClements D.J., Yu Q., Xie J., Zhang H., Huang L., Li B., Tian J., Chen Y. (2025). Anthocyanin-sulfated polysaccharide–ovalbumin nanocomplex: Intestinal absorption mechanism and intracellular antioxidant activity in Caco-2 cells. Food Chem..

[30] Zhang X., Gao X., Yi X., Yu H., Shao M., Li Y., Shen X. (2024). Multi-targeting inulin-based nanoparticles with cannabidiol for effective prevention of ulcerative colitis. Mater. Today Bio.

[31] Zeng L., Peng Q., Li Q., Bi Y., Kong F., Wang Z., Tan S. (2023). Synthesis, characterization, biological activity, and in vitro digestion of selenium nanoparticles stabilized by Antarctic ice microalgae polypeptide. Bioorganic Chem..

[32] Chen Y., Zhu F., Chen J., Liu X., Li R., Wang Z., Cheong K.L., Zhong S. (2024). Selenium nanoparticles stabilized by Sargassum fusiforme polysaccharides: Synthesis, characterization and bioactivity. Int. J. Biol. Macromol..

[33] Liu G., Yang X., Zhang J., Liang L., Miao F., Ji T., Ye Z., Chu M., Ren J., Xu X. (2021). Synthesis, stability and anti-fatigue activity of selenium nanoparticles stabilized by *Lycium barbarum* polysaccharides. Int. J. Biol. Macromol..

[34] Yang Z., Hu Y., Yue P., Tian R., Li H., Lü B., Chen G., Peng F. (2023). Physicochemical stability of lichenan (*Usnea longissima*) decorated-selenium nanoparticles for cancer chemoprevention. Food Biosci..

[35] Su X., Liu W., Yang B., Yang S., Hou J., Yu G., Feng Y., Li J. (2024). Constructing network structures to enhance stability and target deposition of selenium nanoparticles via amphiphilic sodium alginate and alkyl glycosides. Int. J. Biol. Macromol..

[36] Wang X., Liu W., Li Y., Ma L., Lin Z., Xu J., Guo Y. (2023). Preparation and anti-tumor activity of selenium nanoparticles based on a polysaccharide from *Paeonia lactiflora*. Int. J. Biol. Macromol..

[37] Huang Q., Lin W., Yang X.Q., Su D.X., He S., Nag A., Zeng Q.Z., Yuan Y. (2023). Development, characterization and in vitro bile salts binding capacity of selenium nanoparticles stabilized by soybean polypeptides. Food Chem..

[38] Shi M., Deng J., Min J., Zheng H., Guo M., Fan X., Cheng S., Zhang S., Ma X. (2023). Synthesis, characterization, and cytotoxicity analysis of selenium nanoparticles stabilized by *Morchella sextelata* polysaccharide. Int. J. Biol. Macromol..

[39] Zhai C., Lin Y., Mao C., Li X., Zhang R., Liu J., Zhang L. (2024). Construction, characterization, antioxidant activity and effects on properties in vitro digestion of selenium nanoparticles decorated with Cyperus esculentus polysaccharides. Food Biosci..

[40] Zhou L.Z., Song Z.T., Zhang S.J., Li Y.L., Xu J., Guo Y.Q. (2021). Construction and antitumor activity of selenium nanoparticles decorated with the polysaccharide extracted from *Citrus limon* (L.) Burm. f. (Rutaceae). Int. J. Biol. Macromol..

[41] Zhao M., Wu Y., Zhang F., Zheng S., Wang L., Bai J., Yang Y. (2023). Preparation of *Ribes nigrum* L. polysaccharides-stabilized selenium nanoparticles for enhancement of the anti-glycation and α-glucosidase inhibitory activities. Int. J. Biol. Macromol..

[42] El-Din A.S.G.S., Yehia A., Hamza E., A-Elgadir T.M.E., El-Fattah E.E.A. (2024). Selenium nanoparticle ameliorates LPS-induced acute lung injury in rats through inhibition of ferroptosis, inflammation, and HSPs. J. Drug Deliv. Sci. Technol..

[43] Cao B., Zhang Q., Guo J., Guo R., Fan X., Bi Y. (2021). Synthesis and evaluation of *Grateloupia Livida* polysaccharides-functionalized selenium nanoparticles. Int. J. Biol. Macromol..

[44] Zhang J., Yang X., Ji T., Wen C., Ye Z., Liu X., Liang L., Liu G., Xu X. (2022). Digestion and absorption properties of *Lycium barbarum* polysaccharides stabilized selenium nanoparticles. Food Chem..

[45] Tang L., Luo X., Wang M., Wang Z., Guo J., Kong F., Bi Y. (2021). Synthesis, characterization, in vitro antioxidant and hypoglycemic activities of selenium nanoparticles decorated with polysaccharides of *Gracilaria lemaneiformis*. Int. J. Biol. Macromol..

[46] Tao J., Ning W., Lu W., Wang R., Zhou H., Zhang H., Xu J., Wang S., Teng Z., Wang L. (2025). Smart self-transforming nano-systems for overcoming biological barrier and enhancing tumor treatment efficacy. J. Control Release.

[47] Zhang X., Xiao Y., Huang Q. (2023). The cellular uptake of *Cordyceps sinensis* exopolysaccharide-selenium nanoparticles and their induced apoptosis of HepG2 cells via mitochondria- and death receptor-mediated pathways. Int. J. Biol. Macromol..

[48] Wang Y., Chen P., Zhao G., Sun K., Li D., Wan X., Zhang J. (2015). Inverse relationship between elemental selenium nanoparticle size and inhibition of cancer cell growth in vitro and in vivo. Food Chem. Toxicol..

[49] Yu Y.H., Kouame K.J.E.P., Liu X., Yu X., Jin M.Y., Li L.Q., Liu F., Li Y., Yan J.K., Li B. (2025). Preparation, characterization, and induced human colon cancer HCT-116 and HT-29 cell apoptosis performance of selenium nanoparticles stabilized by longan polysaccharides. Int. J. Biol. Macromol..

[50] Zhang X., Xiao Y., Huang Q. (2024). Investigation of cellular uptake and transport capacity of *Cordyceps sinensis* exopolysaccharide-selenium nanoparticles with different particle sizes in Caco-2 cell monolayer. Int. J. Biol. Macromol..

[51] Ouyang J., Deng B., Zou B., Li Y., Bu Q., Tian Y., Tian M., Chen W., Kong N., Chen T. (2023). Oral hydrogel microbeads-mediated in situ synthesis of selenoproteins for regulating intestinal immunity and microbiota. J. Am. Chem. Soc..

[52] Xiao Z., Zhou J., Chen H., Chen X., Wang L., Liu D., Kang X. (2024). Synthesis, characterization and MAFLD prevention potential of *Ganoderma lucidum* spore polysaccharide-stabilized selenium nanoparticles. Int. J. Biol. Macromol..

[53] Wen C., Tang J., Liu D., Fan M., Lin X., Liu G., Liang L., Liu X., Zhang J., Li Y. (2025). Selenium release during the simulated gastrointestinal digestion and antioxidant activity of selenium nanoparticles stabilized by *Grifola frondosa* polysaccharides and gallic acid conjugates. Int. J. Biol. Macromol..

