# Supplementary material for: Poria cocos Polysaccharide-Modified Selenium Nanoparticles: Structural Characterization, Stability, and In Vitro Antioxidant and Anti-Inflammatory Activity Studies
Source: Foods. 2025 Oct 18;14(20):3555. doi: 10.3390/foods14203555 (PMC12563372; doi:10.3390/foods14203555)
Supplement: Supplementary file 1 [file foods-14-03555-s001.zip › foods-3908736-supplementary.pdf]

Supplementary Material

***Poria cocos* polysaccharide-modified selenium nanoparticles: structural characterization, stability, and *in vitro* antioxidant and anti-inflammatory activity studies**

Tao Shu [1, 2], Fan Li [1, 2], Jiang-Ning Hu [3, *] and Yu Xu [1, 2, *]

[1] National Key Laboratory for Development and Utilization of Forest Food Resources, Zhejiang A&F University, Hangzhou 311300, China

[2] College of Food and Health, Zhejiang A&F University, Hangzhou 311300, China

[3] SKL of Marine Food Processing & Safety Control, National Engineering Research Center of Seafood, Collaborative Innovation Center of Seafood Deep Processing, School of Food Science and Technology, Dalian Polytechnic University, Dalian 116034, China

*** To the Correspondence**

Name: Jiang-Ning Hu

E-mail: hujiangning2005@hotmail.com

Mailing address: SKL of Marine Food Processing & Safety Control, School of Food Science and Technology, Dalian Polytechnic University, Dalian 116034, China

Telephone: +86-411-86318731

Name: Yu Xu

E-mail: xuyu@zafu.edu.cn

Mailing address: National Key Laboratory for Development and Utilization of Forest Food Resources, Zhejiang A&F University, Hangzhou 311300, China; College of Food and Health, Zhejiang A&F University, Hangzhou 311300, China

Telephone: +86-15804445204

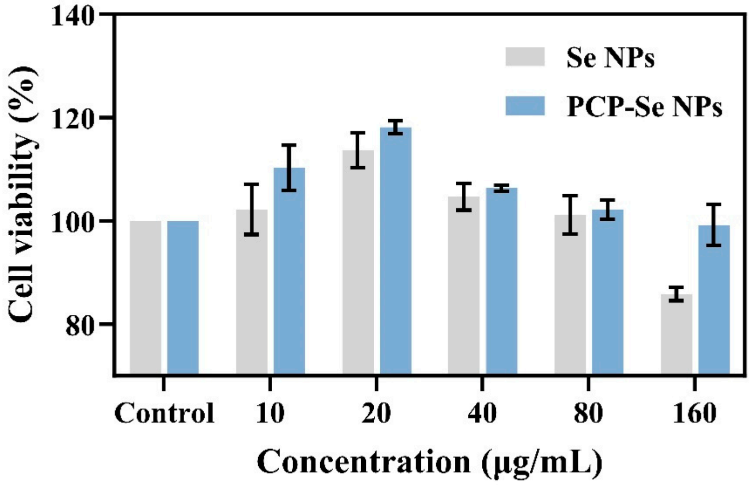

Fig. S1 The effects of different concentrations of Se NPs and PCP-Se NPs on cell viability of RAW264.7 cells.

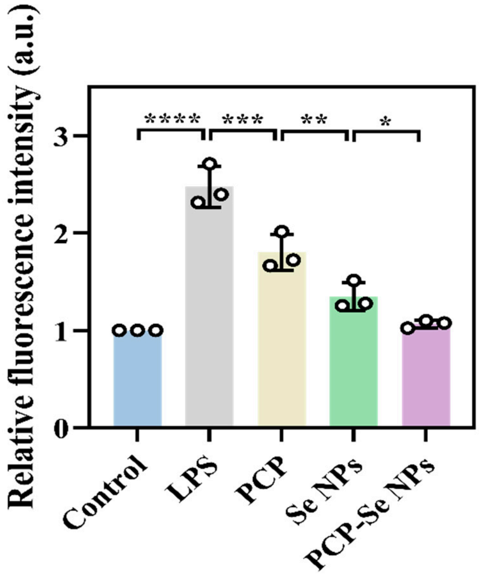

Fig. S2 The relative fluorescence intensity of ROS in RAW264.7 cells treated with PCP, Se NPs and PCP-Se NPs.

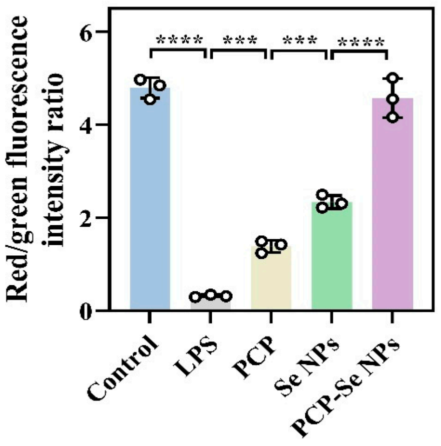

Fig. S3 Red/green fluorescence intensity ratio of mitochondrial membrane potential in RAW264.7 cells treated with PCP, Se NPs and PCP-Se NPs, respectively.
